# The maize B chromosome shapes the transcriptome throughout the entire plant life cycle

**DOI:** 10.1093/jxb/eraf337

**Published:** 2025-07-29

**Authors:** Lucie Hloušková, Zuzana Tulpová, Radim Svačina, Kateřina Holušová, Petr Cápal, Pavla Navrátilová, Miroslava Karafiátová, Jan Bartoš

**Affiliations:** Institute of Experimental Botany of the Czech Academy of Sciences, Centre of Plant Structural and Functional Genomics, Šlechtitelů 31, Olomouc 779 00, Czech Republic; Department of Cell Biology and Genetics, Palacký University, Šlechtitelů 27, Olomouc 779 00, Czech Republic; Institute of Experimental Botany of the Czech Academy of Sciences, Centre of Plant Structural and Functional Genomics, Šlechtitelů 31, Olomouc 779 00, Czech Republic; Institute of Experimental Botany of the Czech Academy of Sciences, Centre of Plant Structural and Functional Genomics, Šlechtitelů 31, Olomouc 779 00, Czech Republic; Institute of Experimental Botany of the Czech Academy of Sciences, Centre of Plant Structural and Functional Genomics, Šlechtitelů 31, Olomouc 779 00, Czech Republic; Institute of Experimental Botany of the Czech Academy of Sciences, Centre of Plant Structural and Functional Genomics, Šlechtitelů 31, Olomouc 779 00, Czech Republic; Institute of Experimental Botany of the Czech Academy of Sciences, Centre of Plant Structural and Functional Genomics, Šlechtitelů 31, Olomouc 779 00, Czech Republic; Institute of Experimental Botany of the Czech Academy of Sciences, Centre of Plant Structural and Functional Genomics, Šlechtitelů 31, Olomouc 779 00, Czech Republic; Institute of Experimental Botany of the Czech Academy of Sciences, Centre of Plant Structural and Functional Genomics, Šlechtitelů 31, Olomouc 779 00, Czech Republic; New Zealand Institute for Plant and Food Research Limited, New Zealand

**Keywords:** B chromosome, gene annotation, gene expression, maize, transcriptome, *Zea mays*

## Abstract

Maize (*Zea mays*) is one of the world’s most important crops and a recognized model for biological research, with some individuals having supernumerary B chromosomes. This B chromosome has been studied for decades, yet its gene expression across different plant tissues has not been thoroughly described. Here, we present a comprehensive transcriptomic atlas of the maize plant with and without the B chromosome. By analysing 11 tissues/organs, we found that genes encoded by the B chromosome contribute to the transcriptome throughout plant growth, with the highest activity observed in reproductive organs. Co-expression analysis revealed a cluster of 30 genes expressed specifically in tassels and indicated that *Shortage in chiasmata 1* is a promising candidate for regulation of crossover frequency mediated by the B chromosome. In addition to its own transcriptional activity, our results also demonstrated that the B chromosome influences the expression of genes located on the A chromosome in all the tissues that we analysed. As well as providing new insights into the expression and regulatory effects of the B chromosome, our study has also generated fundamental information that will provide a basis for exploring its wider biological role.

## Teaser

A new whole-genome assembly of maize identifies a potential regulator of crossover frequency and other effects on expression of A-chromosome genes mediated by the B chromosome.

## Introduction

The genome of maize (*Zea mays*) consists of 10 standard chromosomes, but an additional type, known as the B chromosome, can be found in numerous accessions. B chromosomes have been identified in nearly 3000 plant and animal species (D'Ambrosio *et al*., 2017) since their initial discovery over a century ago (Wilson, 1907). They continue to be a subject of genetic research due to their unique behaviour and potential effects on the host. These supernumerary chromosomes are present in some individuals of a given species but absent in others, and often do not follow a Mendelian inheritance pattern. Instead, they persist in populations through non-traditional transmission mechanisms, which in maize include non-disjunction during second pollen mitosis (Herschel, 1947), preferential fertilization (Herschel, 1948), and increased frequency of univalent transition through meiosis (Carlson and Roseman, 1992).

Unlike the standard set of A chromosomes, B chromosomes are not required for normal development, yet their presence can affect various cellular and physiological processes. At low copy numbers, B chromosomes are usually phenotypically neutral; however, as their copy number increases, their presence can become detrimental (Östergren, 1945; Jones, 1995; Camacho *et al*., 2004; Karafiátová *et al*., 2021). In maize, several effects associated with the presence of B chromosomes have been documented: it increases recombination frequency on A chromosomes (Rhoades, 1968), causes breakages of chromosome with large knobs during the second pollen mitosis (Rhoades *et al*., 1967), results in leaf striping (Staub, 1987), and reduces fertility and vigor when present in high copy numbers (Randolph, 1941).

The structural organization of the maize B chromosome, first identified in the 1920s (Randolph, 1928), sets it apart from the standard genome. It contains four regions of heterochromatin and a distal euchromatin region, a minute short arm, a centromeric knob, and a proximal euchromatin region (Cheng *et al*., 2016), but its composition shares a high degree of similarity with sequences found on the standard A chromosomes (Blavet *et al*., 2021). Blavet *et al*. (2021) published a high-quality sequence of the maize B chromosome, revealing 758 predicted protein-coding genes within a 125.9 Mbp pseudomolecule of the B chromosome. Although initially thought to be transcriptionally inactive, more recent studies have demonstrated that it carries functional genes and can influence the expression of thousands of genes on the standard set of chromosomes (Shi *et al*., 2022). However, most transcriptomic studies have focused on how the B chromosome affects gene activity in leaf samples (Huang *et al*., 2016; Hong *et al*., 2020; Shi *et al*., 2022), and a complete understanding of its expression patterns in individual tissues of the growing plant remains lacking.

Similar effects of the B chromosome on gene expression to those in maize have been observed in lily *Lilium amabile*, where thousands of genes are differentially expressed in plants hosting B chromosomes compared to control plants (Park *et al*., 2019). In the grasshopper *Eyprepocnemis plorans*, the presence of B chromosomes induces changes in gene expression linked to host genome adaptation (Navarro-Domínguez *et al*., 2019). Studies in rye (*Secale cereale*) and the cichlid fish *Astatotilapia latifasciata* have further revealed that B chromosomes encode functional proteins or transcribe non-coding RNAs, indicating their potential regulatory roles (Ma *et al*., 2017; Ramos *et al*., 2017).

The unique properties of the maize B chromosome provide a promising platform for engineering minichromosomes. Such artificial chromosomes can be designed to carry specific gene constructs without disrupting the native genome. For instance, the concept of using B-derived minichromosomes for transgene stacking or introduction of traits has been proposed and explored (e.g. see reviews by Houben and Schubert, 2007; Birchler *et al*., 2016, 2024). Although such applications are still at the concept stage, understanding B chromosome biology lays the groundwork for developing tools for targeted gene regulation, stress resilience, and synthetic chromosome systems for crop improvement.

In this study, we examined the transcriptomic activity of the B chromosome in a wide array of maize tissues. Our resulting construction of a comprehensive transcriptomic atlas provides a broader view of the expression of genes encoded by the B chromosome and facilitates a deeper understanding of its effect throughout the entire life cycle of maize. The resources we have developed in this study will support further detailed analyses on the effects of the presence of the B chromosome in maize.

## Materials and methods

### Plant material and growing conditions

The maize (*Zea mays*) inbred line containing the B chromosome was kindly provided by Dr James A. Birchler (University of Missouri, Columbia, USA), whilst the reference B73 inbred line without B chromosomes was obtained from the Plant Introduction Station (North Central Regional Plant Introduction Station, Iowa, USA).

Plants the inbred line B73 containing two B chromosomes were used for whole-genome sequencing, high-throughput chromatin conformation capture, Bionano optical mapping, and RNA-sequencing analysis. Maize seeds were germinated at 25 °C for 5 d. A direct PCR approach was used to assess the presence of the B chromosome in individual seedlings. The seedlings with the B chromosome were further scored using a droplet digital PCR (ddPCR) protocol to estimate the number of B chromosomes (Svačina *et al*., 2023). The selected plants were then grown in a greenhouse under controlled conditions of 14/10 h light/dark (250 μmol m^–2^ s^–1^) at 24/20 °C. The developmental stages of the plants were determined using leaf collar method (Begcy and Dresselhaus, 2017).

For RNA-seq analysis, 11 tissue types from various developmental stages were collected (Supplementary Fig. S1), immediately frozen in liquid nitrogen, and then stored at −80 °C. The tissues examined were as follows: whole germinated seedling (WS), the primary root (PR), and first leaves (PL) at the one-leaf stage (V1); first stem node (FN) and leaf tip of the youngest actively growing leaf (ST) at the five-leaf stage (V5); meiotic tassel (MT); immature tassel (IT); anthers with mature pollen (ANT); unpollinated immature cob (IMMC); and the husk (HUSK) and silk (SILK) of the unpollinated immature cob. For early tissues such as WS and the V1 tissues, the number of maize B chromosomes was determined using fluorescence *in situ* hybridization (FISH) with a B-specific repeat on root meristems (as described by Karafiátová *et al*., 2021; Svačina *et al*., 2023). Four biological replicates were collected for each tissue for both the control B73 inbred line without B chromosomes and the B73 inbred line containing two B chromosomes.

### Nanopore sequencing

The library for sequencing was prepared based on the protocol of the Rapid Sequencing Kit SQK-RAD004 (Oxford Nanopore Technologies, UK, version RSE_9046_v1_revM_14Aug2019), with 1000 ng of maize high-molecular-weight (HMW) DNA as input material in 7.5 µl of Tris. This was mixed with 2.5 µl of fragmentation mix (FRA) and incubated at 30 °C for 1 min and then at 80 °C for 1 min. The sample was then cooled on ice. The library was finished by adding 1 µl of Rapid Adapter (RA) followed by 5 min of incubation at room temperature.

The library for ultra-long sequencing was prepared based on the protocol of the Ultra-Long DNA Sequencing Kit SQK-ULK001 (Oxford Nanopore Technologies, version: ULK_9124_v110_revE_24Mar2021), except that we replaced the isolation step with the following procedure. HMW DNA was extracted from leaf nuclei isolated using a protocol adapted from Vondrak *et al*. (2020). Six batches of fresh young leaf tissue (5 g each) were homogenized using a mortar and pestle in liquid nitrogen, transferred into beakers on ice containing 35 ml of Buffer H (10 mM Tris, 80 mM KCl, 10 mM EDTA, 4 mM spermidine, 1 mM spermine, 0.5 M sucrose, 0.5% Triton X-100, 0.1% mercapthoethanol, pH 9.4), incubated at room temperature for 5 min, and filtered through a 50 µm nylon mesh. The samples were then repeatedly centrifuged for 15 min at 400 *g* at 4 °C, and the pellets were resuspended in 35 ml of Buffer H until the supernatants turned yellow. Pellets of three tubes were resuspended and combined in 20 ml of ice-cold TC buffer (50 mM Tris, 75 mM NaCl, 6 mM MgCl_2_, 0.1 mM CaCl_2_, pH 7.5). The samples were centrifuged for 5 min at 400 *g* and 4 °C and the pellet was resuspended in 3 ml of TC buffer. Then, 30 µl of proteinase K (10 mg ml^–1^), 600 µl of mercaptoethanol, and 3.6 ml of 2X CTAB (1.4 M NaCl, 100 mM Tris, 2% CTAB, 20 mM EDTA, 2% polyvinylpyrrolidone, 0.5% NaHSO_3_, pH 8) was added to each sample and incubated at 37 °C for 20 min, with gentle mixing twice during this period. The samples were then purified using chloroform:isoamyl alcohol (24:1) and the upper phase from all tubes was transferred to a single 50 ml tube. DNA was precipitated by adding 60% of the sample volume of isopropanol, and the precipitate was washed with ice-cold 70% ethanol and then dissolved in 750 µl EEB (10 mM Tris, 1 mM EDTA, 0.5% Triton X-100, pH 9). The library was prepared using 30–40 µg of HMW DNA in 750 µl EEB, starting from the tagmentation step of the Ultra-Long DNA Sequencing Kit SQK-ULK001 protocol, as follows. Diluted FRA was prepared by mixing 6 µl of FRA and 244 µl of FRA Dilution Buffer. The diluted FRA was added to the HMW DNA sample and mixed using a wide-bore pipette tip, after which it was incubated at room temperature for 5 min and then at 75 °C for 5 min. After cooling the reaction at room temperature for 10 min, 5 µl of RA was added and mixed using a wide-bore pipette tip. The DNA was precipitated by adding 1 ml of PPT (1 ml of deionized water, 3.4 mg spermine) and mixing by turning upside down several times. After formation of a glassy white mass, the sample was centrifuged at 1000 *g* for 1 min. The supernatant was removed, and the sequencing library was dissolved in 225 µl of elution buffer and incubated at room temperature overnight, for at least for 12 h. Library loading on a R9.4.1 flow cell (Oxford Nanopore Technologies) was performed based on the manufacturer’s instructions for all libraries. Basecalling of the raw sequencing data in fast5 format was performed using Guppy v6.1.5-GPU (Wick *et al*., 2019).

### Whole-genome sequencing

Genomic DNA was isolated from 100 mg of leaf material using a NucleoSpin Plant II Kit (Macherey-Nagel) according to the manufacturer’s instructions. Libraries for sequencing were prepared using a NEBNext Ultra II DNA Library Prep Kit for Illumina (#E7645L, New England Biolabs), and were sequenced on an Illumina NovaSeq 6000 instrument with 2×250 bp paired-end reads, producing almost 270 million paired-end reads in total.

### High-throughput chromatin conformation capture library preparation and sequencing

High-throughput chromatin conformation capture (Hi-C) was performed on 4-day-old maize seedlings. Seedlings were fixed in 2% formaldehyde in 10 mM Tris (pH 8) under vacuum for 15 min. Fixation was stopped by 1.25 mM glycine in 1× PBS buffer for 5 min under vacuum, and the material was washed three times in cold 1× PBS before nuclei extraction. Fixed nuclei were released by mechanical chopping using a razor blade into LB01 buffer and stained by DAPI at a final concentration of 2 μg ml^–1^. Two replicates of Hi-C libraries were prepared. For each replicate, five million nuclei were flow-sorted (Šimková *et al*., 2003) on a FACSAria SORP flow cytometer (Becton Dickinson, San Jose, CA USA) into a 15 ml Falcon tube with 2 ml LB01 buffer (Doležel *et al*., 1989), centrifuged at 500 *g* for 30 min at 4 °C and the supernatant was removed except for 20 μl. The pelleted nuclei were gently resuspended, and the entire sample was transferred to a 1.5 ml Eppendorf tube. The Hi-C library preparation was carried out with an Arima-HiC Kit (Arima Genomics, A510008) according to the manufacturer’s protocol (A160432 v01). The sequencing libraries were prepared with a NEBNext Ultra II DNA Library Prep Kit for Illumina (#E7645L, New England Biolabs) with 10 cycles of PCR amplification. Libraries were sequenced on an Illumina NovaSeq 6000 instrument, with 2× 150 bp paired-end reads, producing almost 337 million paired-end reads in total.

### Sequence assembly and scaffolding

For *de novo* whole-genome assembly, Canu assembler was used (Koren *et al*., 2017) with corMhapOptions set to ‘--num-min-matches 10 --repeat-idf-scale 50’ to reduce the effects of repeats on disk space used and to speed up the run. The output assembly from Canu was polished by two iterations of NextPolish (Hu *et al*., 2020) with default settings using Illumina reads. The assembly was then scaffolded using Hi-C data via YaHS (Zhou *et al*., 2023) with range of resolution set to 10 kb–5 Mb (–r 10 000, 20 000, 50 000, 100 000, 200 000, 500 000, 1 000 000, 2 000 000, 5 000 000).

### Bionano optical map construction

HMW DNA was prepared for optical mapping. A total of one million nuclei were purified by flow cytometry from the B73 inbred line containing two B chromosomes. The nuclei were embedded in agarose plugs and treated with proteinase K as described by Šimková *et al*. (2003). Then, 540 ng of released HMW DNA were directly labelled at DLE-1 recognition sites (CTTAAG motif) following a standard Bionano Prep Direct Label and Stain (DLS) protocol (Bionano Genomics, San Diego, USA), and analysed on the Saphyr platform of Bionano Genomics using Saphyr chip G1.2. The 1381.1 Gbp of data collected for single molecules >150 kbp were filtered based on molecule maximum fluorescence, which resulted in a dataset consisting of 947.5 Gbp of molecules with maximum fluorescent intensity of 2000 and a N50 value (a weighted average length of DNA molecules in the dataset) of ∼255.5 kbp, corresponding to 421× effective coverage of the B73 genome (including B chromosome). The dataset was further used to generate *de novo* assembly using the Bionano Solve software (v. 3.6.1_11162020) using the parameters ‘*optArguments_nonhaplotype_noES_noCut_DLE1_saphyr.xml*’. The *de novo* assembly was aligned to the *Zea mays* reference genome assembly RefGen_v5 (Hufford *et al*., 2021) using the Access software v. 1.7.1 (Bionano Genomics), and the optical map contigs that were not aligned were considered to be putative B-originated contigs and used for scaffolding validation and further gap closing.

### Gap-closing and assembly evaluation

To resolve gaps within Hi-C scaffolds, we used an approach combining optical maps, contigs from Canu, and Oxford Nanopore Technology (ONT) reads, which were aligned to the Hi-C scaffolds in the Access software v. 1.7.1. Gaps without any evidence for closing were left in the final sequence, represented by 200 Ns. For some Hi-C gaps between two correctly placed contigs, no gap existed in the genome sequence (i.e. the optical map and ONT reads aligned perfectly to the reference sequence), and such gaps were closed in the final sequence. Additionally, in specific cases (Supplementary Fig. S2), two contigs had duplicated ends but were joined by Hi-C without modification. We removed one copy of artificially duplicated region based on optical maps, thereby closing the gap. These modifications were all manually curated using Geneious version 7.1.2 (www.geneious.com). The final assembly was polished by three iterations of NextPolish with default settings using the ONT reads followed by three iterations of NextPolish using the Illumina reads. The completeness of the *de novo* assembly was evaluated using the Benchmarking sets of Universal Single-Copy Orthologs (BUSCO) v.5.4.5 program (Simão *et al*., 2015) based on the OrthoDB v10 *Poales* database. Synteny and structural rearrangements were computed using SyRI (Goel *et al*., 2019) and visualized using plotsr (Goel and Schneeberger, 2022).

### RNA extraction and sequencing

Total RNA was isolated from four replicates of each of the 11 tissues of the control B73 inbred line (no B chromosome) and the B73 inbred line containing two B chromosomes using a Spectrum Plant Total RNA Kit (Sigma-Aldrich), following the manufacturer’s instructions. The quality of the total RNA was measured using an Agilent 2100 Bioanalyzer and construction of the indexed sequencing libraries was performed using a NEBnext Ultra II RNA Library Prep Kit for Illumina according to the manufacturer's protocol. Libraries were sequenced on an Illumina NovaSeq 6000 instrument, with 2× 150 bp paired-end reads, producing ∼36 million paired-end reads on average.

### Gene prediction and functional annotation

As an input for the genome annotation pipeline, we used soft-masked genome sequence from RepeatMasker (Smit et al., 2013-2015). For masking we used Maize TE Consortium (MTEC) curated library of repeats (https://github.com/oushujun/MTEC) and a custom-made library of repeats of the B chromosome sequence generated using DANTE and DANTE_LTR (Novák *et al*., 2024). The B chromosome sequence (extracted from the assembly) was merged with the *Z. mays* reference RefGen_v5 sequence, with prior repeat masking. A fully automatic BRAKER3 annotation pipeline was used for prediction of genes (Gabriel *et al*., 2024). To support gene predictions, we used our transcriptomics data from the 11 maize tissues together with the protein sequences of the *Poaceae* family from OrthoDB v11 (Kuznetsov *et al*., 2023). Within the pipeline, AUGUSTUS was set to use also *ab initio* prediction. At the end of the pipeline, the UTR sequences were annotated based on the transcriptomes provided. In addition to the gene models identified in BRAKER3, we supplemented the annotation manually with genes from a previously published sequence of the maize B chromosome (Blavet *et al*., 2021) whenever possible. Further, we evaluated gene models and manually corrected some of them based on alignment of the transcriptomics data. Gene models overlapping with known repetitive sequences were deleted. The confidence class of gene models was established using blastx from the Blast+ package (Camacho *et al*., 2009) based on the homology. First, the *Zea mays* reference RefGen_v5 protein sequence was used as database and, second, blastx was performed with a database containing validated proteomes of *Magnoliopsida* downloaded from Uniprot (https://www.uniprot.org/). Best hits were selected for each predicted gene model in each search. Gene models with query coverage >50% and subject coverage >50% in any of the two blastx searches were considered high-confidence genes. Predicted genes with subject and query coverage below 50% in both searches, or with stop codons in their coding sequences, or without known position of the start codon were classified as low-confidence genes. Functional gene annotation was performed using the Blast2GO package (Conesa *et al*., 2005) on mRNA sequences. Homologs between genes from the largest identified chromosome B scaffold and the 10 chromosomes of the *Z. mays* reference RefGen_v5 were visualized using Circos (Krzywinski *et al*., 2009). Gene density was plotted using the karyoploteR (v.1.32.0) package in R (Gel and Serra, 2017).

### RNA-seq analysis

All RNA-seq reads were pre-processed for quality control with FastQC (v.0.12.1; https://www.bioinformatics.babraham.ac.uk/projects/fastqc/). The fastp software (Chen *et al*., 2018) was applied to filter out sequencing adaptors and for trimming low-quality sequences (parameters: -q 30, -w 4, -l 50). Trimmed reads were mapped using STAR (Dobin *et al*., 2013) to the *Z. mays* reference genome RefGen_v5 supplemented with the reference of the maize B chromosome generated in this study (designated as Maize_Bchr_v2.0). Mapped reads were counted using RSEM (Li and Dewey, 2011). Transcripts per million (TPM) was calculated using the Tximport (v.1.34.0) package in R (Soneson *et al*., 2015) and principal component analysis and analysis of differential expression were performed using the DESeq2 (v.1.12.3) package in R (Love *et al*., 2014). Genes with *P*-value <0.05 and |log_2_(fold-change)| >2 were considered to be significantly differentially expressed genes.

### Gene set Enrichment analysis

Gene-set enrichment analysis (GSEA) was performed using the topGO (v.2.58.0) package in R (http://dx.doi.org/10.18129/B9.bioc.topGO), using gene ontology (GO) terms provided for the *Z. mays* reference RefGen_v5 downloaded from MaizeGDB (https://www.maizegdb.org/) together with GO terms obtained using the Blast2GO pipeline for the genes on the B chromosome. GO terms were considered significantly enriched for *P*<0.05 using Fisher’s exact test. GSEA was performed on a subset of B chromosome-located genes with TPM>1, on a subset of differentially up-regulated genes located on the A chromosomes, and on all genes annotated on the B chromosome in comparison to genes on the A chromosomes.

### Co-expression analysis

For weighted gene co-expression network analysis (WGCNA), a gene co-expression network was constructed for, first, only genes located on the B chromosome with a sum of TPM values in all tissue samples >1 (569 genes), and second, genes located both on the B chromosome and A chromosomes with sum of TPM values in all tissue samples >1 (36 482 genes). Raw read counts were transformed by variance stabilizing transformation using the DESeq2 v.1.12.3 package in R. The soft-thresholded Pearson correlation function in the WGCNA package (v1.53; Langfelder and Horvath, 2008) was used to determine the optimal soft-thresholding power for network construction, with power = 4 for both datasets. Genes with highly similar co-expression patterns were clustered using hierarchical clustering. Modules were identified using the dynamic hybrid tree cut algorithm (Langfelder and Horvath, 2008) and were represented by distinct colour codes, with six modules for genes located on the B chromosome, and 169 modules for genes located on both the B and A chromosomes. Genes that did not cluster into any defined module were assigned to a ‘grey’ module, which was excluded from subsequent analyses.

## Results and discussion

### The new pseudomolecule assembles nearly complete maize B chromosome

**Fig. 1.**
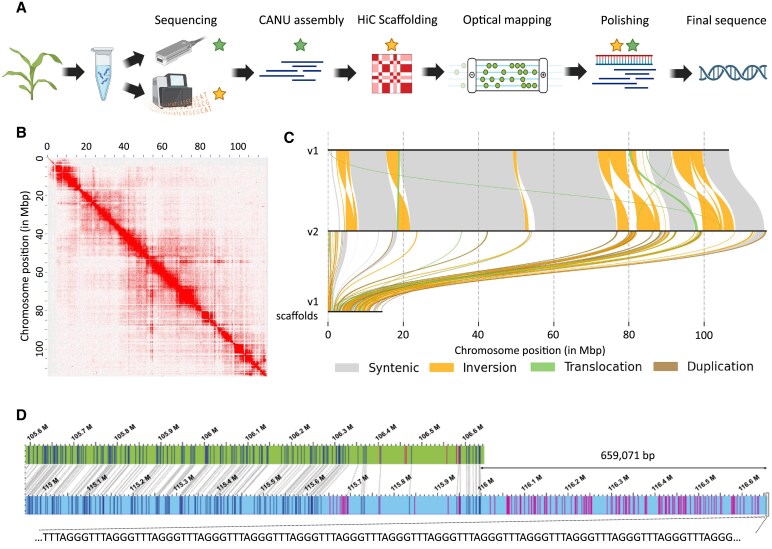
Constructing a new reference sequence of the maize B chromosome. (A) Schematical illustration of the steps towards generation of the new reference sequence of the maize B chromosome. Green stars indicate the use of Oxford Nanopore long reads and yellow stars the use of short reads from Illumina sequencing in particular steps. (B) High-throughput chromatin conformation capture (Hi-C) contact map of the final B chromosome pseudomolecule. The intensity of the red colour corresponds to the frequency of contacts between two particular chromosomal regions in the 3D organization of the chromatin in the nucleus; both axes represent the same pseudomolecule of the B chromosome generated in this study. (C) Visualization of synteny and structural rearrangements of the reference sequence between the version of the pseudomolecule generated by Blavet *et al*. (2021) (v1; Zm-B73_B_CHROMOSOME-MBSC-1.0) and the *de novo* version generated in this study (v2). The synteny shows inversions (orange), small translocations (green), and large blocks of syntenic regions (grey). The synteny between v1 scaffolds and v2 indicates the incorporation of short scaffolds from v1 into the new v2 sequence with duplications shown in brown. (D) Comparison of the telomeric region of the long arm of the B chromosome pseudomolecule based on *DLE-1* enzyme sites in the version generated by Blavet *et al*. (2021) (top) and the *de novo* version generated in this study (bottom). Matching sites are represented by blue vertical lines, while sites missing in the other pseudomolecule are represented by pink lines. The length of the telomeric region added to the end of the pseudomolecule in this study is indicated, including a 7.5 kb array of telomeric repeat.

A total of 43× sequence coverage of raw Oxford Nanopore Technology (ONT) data (Supplementary Table S1), 28× coverage of Illumina data, and an additional 21× coverage of Hi-C data was generated for the *de novo* assembly of the maize genome (Fig. 1A). The initial ONT assembly with Canu generated 4290 contigs with a total length of 2852 Mb and an N50 value of 3.7 Mb. The assembly was then polished using Illumina sequencing data and scaffolded using Hi-C data (Fig. 1B. The resulting whole-genome assembly, which included both the standard A chromosomes and the B chromosome, comprised 3599 scaffolds with a total length of 2.86 Gb (Table 1). Alignment of the scaffolds to the *Z. mays* reference RefGen_v5 demonstrated the high quality of the *de novo* assembly. Four chromosomes (2, 6, 9, and 10) were each fully covered by a single scaffold, while chromosomes 5 and 7 were represented by two scaffolds each. These results illustrate the high accuracy of our Hi-C-based scaffolding. The N50 for the final assembly (152.7 Mb) increased by ∼40-fold compared with the initial assembly. The completeness of the final assembly was 98.1%, as measured by the BUSCO score (Supplementary Table S2), again confirming the high quality of the genome-wide assembly.

**Table 1.** Summary statistics for the *de novo* assembly generated in this study

**Whole genome**	
Assembly size (bp)	2 863 921 841
Number of scaffolds	3599
Longest scaffold (bp)	241 399 949
Scaffolds N50	152 709 489
**B chromosome**	
Assembly size (bp)	134 331 364
Number of scaffolds	60
Longest scaffold (chrB) (bp)	116 657 383
Scaffolds N50 excluding chrB	570 773

From the initial assembly, 56 contigs were identified as originating from the B chromosome, using k-mer profiling as described in Blavet *et al*. (2021). These contigs totaled 134.3 Mb. After Hi-C scaffolding, 61 B chromosome-derived scaffolds were obtained. The largest of these scaffolds (designated as chrB) represented 116.8 Mb, while the N50 for the remaining B scaffolds was 569 kb (Supplementary Table S3). For gap-closing, we used a strategy that combined optical maps (Supplementary Table S4), Canu-assembled contigs, and long ONT reads. This manual curation was used exclusively for B-originating scaffolds and successfully resolved 9 out of 23 gaps. Finally, the whole genome assembly was polished using both ONT and Illumina reads. The final assembly of the B chromosome had a cumulative size of 134.3 Mb (Table 1). The improved, nearly telomere-to-telomere, B-chromosome pseudomolecule (i.e. the longest B-originating scaffold, chrB) had a total length of 116 657 383 bp.

This new reference pseudomolecule offered significant improvements compared with the previously published sequence (Blavet *et al*., 2021). The orientation of some regions was corrected (Fig. 1C; see also Supplementary Fig. S3 for comparison), as further confirmed by optical mapping. Additionally, the 7.8 kb telomeric repeat array of the short arm and the 7.5 kb telomeric repeat array of the long arm, which were missing in previous version, were included in the new reference sequence (Fig. 1D). Beyond the pseudomolecule, the new reference sequence also included 59 additional scaffolds, named as scaffold_0001 to scaffold_0059, with 17.7 Mb size in total and N50 of 571 kb. These scaffolds could not be incorporated into the pseudomolecule due to the high repeat content. Despite this limitation, the total size of the updated reference sequence was increased from 125.9 Mb (Blavet *et al*., 2021) to 134.3 Mb. This revised size more closely approximates the predicted 141 Mb length of the B chromosome, as previously estimated by flow cytometry (Blavet *et al*., 2021).

### The maize B chromosome contains 689 protein-coding genes

**Fig. 2.**
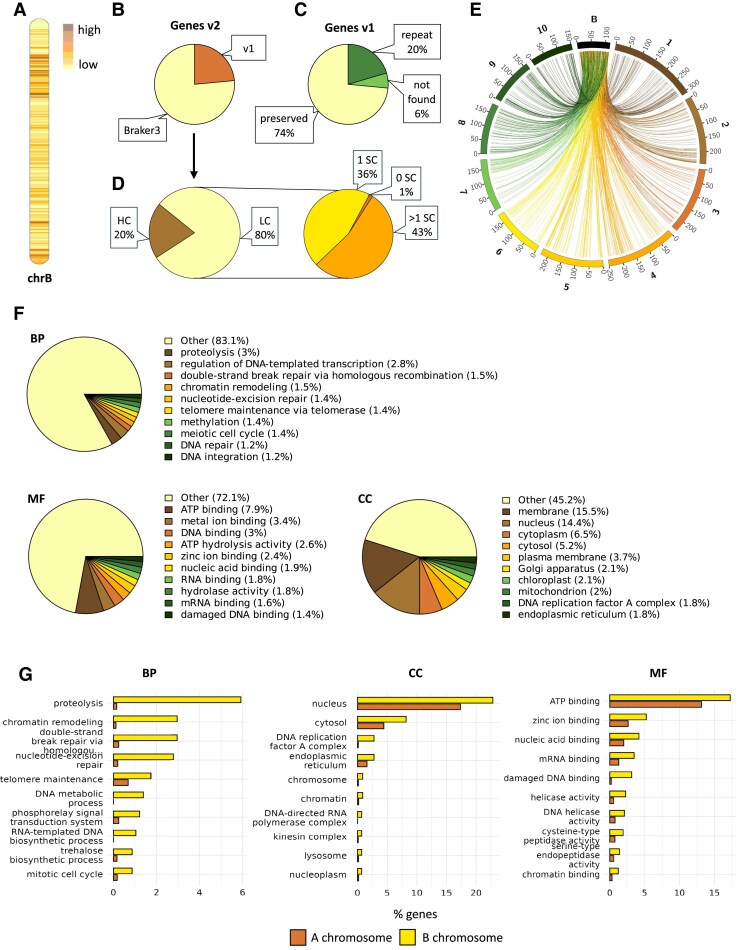
Genes annotated on the *de novo* maize B chromosome reference sequence. (A) Ideogram of gene density of the pseudomolecule, as indicated by the key. (B) Origin of the gene annotation in the *de novo* reference (v2): BRAKER3 indicates gene models predicted by the BRAKER3 pipeline and v1 indicates transfer from the reference sequence generated by Blavet *et al*. (2021). (C) Classification of gene models from the Blavet *et al*. (2021) reference (v1) with respect to the *de novo* annotation: preserved, original gene models having homologs in v2; repeat, gene models removed from the annotation due to overlap with repeats; not found, gene models with no homologs in v2. (D) Classification of gene models into high-confidence (HC) and low-confidence (LC) categories. SC, stop codon. (E) Visualization of homology between genes from the v2 pseudomolecule and 10 chromosomes of the *Zea mays* reference RefGen_v5. Each ribbon corresponds to a homolog pair, and numbers correspond to Mb positions. (F) Functional classification of the maize B chromosome representing the distribution of genes based on their GO annotations of biological processes (BP), molecular functions (MF), and cellular component (CC). Only the 10 GO terms with the highest representation are shown for each category, and their proportions relative to all genes are shown. (G) GO enrichment analysis of the B chromosome genes. Only the 10 GO terms with the highest percentage of B chromosome-encoded genes were selected for visualization. The GO terms are significantly over-represented in the B chromosome compared with the A chromosomal complement (Fisher's exact test, *P*<0.05).

Gene annotation was performed using the BRAKER3 pipeline and supplemented with gene models from the previous version published by Blavet *et al*. (2021), hereafter referred to as ‘v1’. This resulted in the prediction of 689 protein-coding genes on the maize B chromosome (Fig. 2; Supplementary Dataset S1). The average gene length was 1672 bp with the average length of the coding DNA sequence (CDS) being 1049 bp. Of the predicted genes, 527 gene models (76%) were derived from the BRAKER3 pipeline, while the other 162 (24%) were transferred from the previous v1 version of the sequence (Fig. 2B). In comparison with v1, which has 758 annotated gene models, we retained 557 genes (74%) and discarded 155 (20%) due to their overlap with repetitive/transposable elements. The remaining 46 genes (6%) from v1 were not found in our new annotation (Fig. 2C; Supplementary Dataset S2). As a result of the new annotation pipeline BRAKER3 and using a masked sequence as an input, our revised annotation includes only four transposable element genes, reflecting a significant improvement in accuracy and specificity.

The protein-coding genes were further categorized into two groups, those with high-confidence (HC) and low-confidence (LC) (Fig. 2D), based on their sequence similarity to proteomes from the *Magnoliopsida* class and the *Z. mays* RefGen_v5 protein sequences (Fig. 2E; Supplementary Dataset S3). A total of 137 gene models (20%) were classified as HC, while the remainder were categorized as LC for one of three reasons: either the sequence similarity with known proteins was <50%, there was an absence of a defined start or stop codon, or there was a presence of more than one stop codon in the coding sequence. Among the LC gene models, 296 were found to contain multiple stop codons, most likely due to frameshift mutations or other evolutionary changes that disrupted the coding sequences.

We performed a GO analysis using the Blast2GO software to investigate the functional roles of 689 annotated genes encoded by the B chromosome. For 574 genes (83.3%) at least one GO term was assigned (Supplementary Dataset S1). Additionally, 115 genes were not annotated with any GO term in the first phase that utilized a blast search (Camacho *et al*., 2009) against the NCBI database (https://www.ncbi.nlm.nih.gov/). Nevertheless, 92 of these genes were annotated in the second phase using an InterPro similarity search. Among the biological processes, the most prevalent GO term was ‘proteolysis’ (GO:0006508), followed by ‘regulation of DNA-templated transcription’ (GO:0006355), ‘double-strand break repair via homologous recombination’ (GO:0000724), and ‘chromatin remodelling’ (GO:0006338) (Fig. 2F). In the molecular function category, the most represented GO term was ‘ATP binding’ (GO:0005524), followed by ‘metal ion binding’ (GO:0046872) and ‘DNA binding’ (GO:0003677). In the cellular component category, ‘membrane’ (GO:0016020) was the most abundant GO term, followed by ‘nucleus’ (GO:0005634) and ‘cytoplasm’ (GO:0005737).

We then performed GO enrichment analysis. In comparison to the A chromosomal complement, 85 GO terms are over-represented in the B chromosome sequence (Fisher’s exact test *P*<0.05; Fig. 2G; Supplementary Dataset S4), which was consistent with the results of Blavet *et al*. (2021). Also consistent with that study, similar GO terms were found to be over-represented such as ‘proteolysis’ (GO:0006508), ‘ATP binding’ (GO:0005524), ‘microtubule cytoskeleton organization’ (GO:0000226), ‘microtubule motor activity’ (GO:0003777) and ‘mitotic cell cycle’ (GO:0000432). Interestingly, the list of GO terms enriched in the sequence of the B chromosome contained multiple GO terms related to chromatin, including ‘chromatin remodelling’ (GO:0006338), ‘chromatin organization’ (GO:0006297), and ‘chromatin binding’ (GO:0003682), which indicated the activity of the B chromosome with respect to its specific inheritance.

### The footprint of B chromosome expression is evident throughout the life of the plant

Previous studies have demonstrated that the maize B chromosome contains actively transcribed genes in leaves (Huang *et al*., 2016; Hong *et al*., 2020; Shi *et al*., 2022). To expand our knowledge, we further explored the patterns of B-chromosome gene expression in selected tissues across the whole anatomy and the life span of the plant.

For a comprehensive transcriptome analysis, RNA-seq was performed for 11 different tissues across the life span of plants of the maize inbred line B73 with no B chromosome and a B73 inbred line with two B chromosomes (Supplementary Fig. S1). On average, 36 million reads were generated per sample, and they were mapped to reference sequence with a success rate 90.8%.

**Fig. 3.**
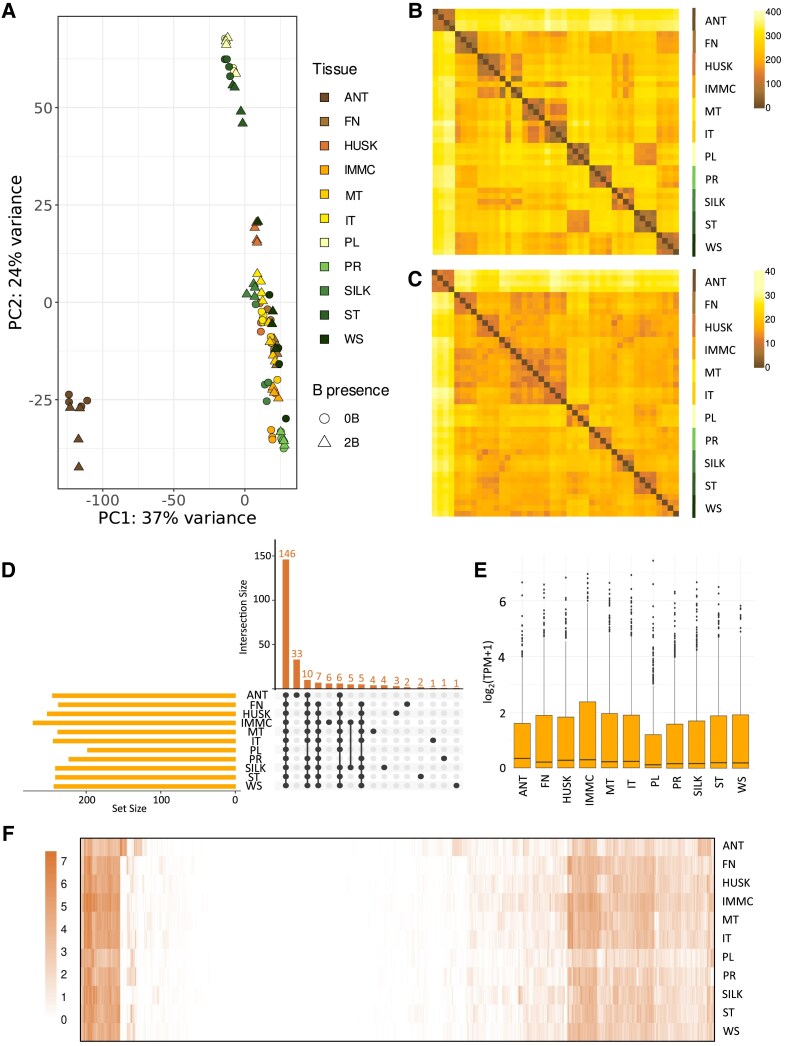
Expression of genes encoded by the maize B chromosome in different tissues at different plant development stages, as determined in a B73 inbred line containing two B chromosomes. (A) Principal component (PC) analysis of global gene expression patterns in the B73 inbred line without B chromosomes (0B) and a B73 inbred line containing two B chromosomes (2B). ANT, anthers with mature pollen; FN, first stem node; HUSK of the unpollinated immature cob; IMMC, unpollinated immature cob; MT, meiotic tassel; IT, immature tassel; PL, first leaves at the one-leaf stage; PR, primary root at the one-leaf stage; SILK, silk of the unpollinated immature cob; ST, leaf tip of the youngest actively growing leaf at the five-leaf stage; WS, whole germinated seedling (Supplementary Fig. S1). (B, C) Similarity matrices of samples based on the expression of (B) genes encoded by the A chromosomes and (C) genes encoded by the B chromosome. In both matrices (B, C), samples have the same order in rows and columns. The colour indicates pairwise Euclidian distance between samples as determined based on the TPM values (Supplementary Dataset S5). (D) UpSet plot showing the number of genes located on the B chromosome with TPM>1 across different tissues of the 2B plants. Only intersections containing at least five genes and those unique to a single tissue are displayed. (E) Box-plot of log_2_(TPM+1) values for each tissue. Boxes were constructed based on all genes annotated on the B chromosome; each box represents expression in a particular tissue. (F) Heatmap constructed based on normalized TPM values. Each row corresponds to a different tissue type and each vertical line represents an individual gene. Expression is represented in log_2_(TPM+1) values, according to the key.

Principal component (PC) analysis showed a good relationship among the samples of the individual tissues (Fig. 3A). The samples from anthers containing mature pollen (ANT) formed a distinct cluster separated from the other tissues, and PC1 primarily explained this divergence. PC2 captured differences between leaf stages (first leaves, PL, and young leaf at the five-leaf stage, ST) and the other tissues. However, there was some overlap and loose clustering among samples of some tissues, particularly in reproductive tissues, both the male tassels (meiotic and immature tassels, MT and IT, and ANT) and female ears (unpollinated immature cob, IMMC, and its SILK and HUSK). We next generated phylogenetic heatmaps, where pairwise similarity between samples was determined by Pearson correlation of the TPM values (Supplementary Dataset S5). Prior to analysis, genes were sorted into two groups based on their chromosomal location. Analysis based on A- and B-chromosomal genes both showed that expression in ANT remained significantly distinct from the other tissues (Fig. 3B, 3C). However, we also observed some clustering among samples of cob tissues and early tassel tissues. This was more notable for genes on the B chromosome compared with those on the A chromosomes, where the clusters were more clearly defined.

We next performed an expression analysis to compare tissues of plants with the B chromosome, with particular focus on genes encoded by that chromosome. Out of 689 genes predicted to be located on the B chromosome, we identified 352 transcribed genes that had TPM>1 in at least one of the examined tissues (Fig. 3D, 3F). Notably, 146 of these were found to be transcribed uniformly across all the tissues. This adds to the results of Shi *et al*. (2022), who reported that 273 genes located on the B chromosome are actively transcribed. Having analysed a wider selection of tissues across different growth stages, our results show that B-chromosomal transcriptional activity is continuous through the whole life-cycle of the maize plant. The expression of B-encoded genes was most prevalent in reproductive organs, with the highest number observed in the immature cob (271), followed by the husk (251), anthers (244), and immature tassel (243). The median log_2_(TPM+1) values for all the genes located on the B chromosome were highest in anthers [median log_2_(TPM+1)=0.26], followed by the immature cob (0.22), and the husk (0.21) (Fig. 3E). These results collectively suggest that the B chromosome is most active in reproductive tissues, implying a possible functional role of its genes in the development and maturation of these organs, and in its transmission to the next generation.

To further investigate expression of the genes on the B chromosome, we performed a gene-set enrichment analysis (GSEA) of genes with TPM>1 using the TopGO software, using our B-chromosome GO annotation (Supplementary Dataset S1) as reference database. Fisher’s exact test (*P*<0.05) identified 41 GO terms that were significantly enriched for at least one tissue (Supplementary Fig. S4; Supplementary Dataset S6). Notably, no significant enrichment was observed for biological processes, molecular functions, or cellular components in the analysis of anthers with mature pollen (ANT), whilst for pooled leaf samples (ST, PL), no biological process GO terms reached significance.

In 8 out of the 11 tissues examined, GO terms related to microtubule functions were significantly enriched, such as ‘microtubule-based process’ (GO:0007017), ‘cytoskeleton organization’ (GO:0007010), and ‘microtubule cytoskeleton organization’ (GO:0000226). This trend extended to the cellular component category, where ‘cytoskeleton’ (GO:0005856) was enriched in 10 out of 11 tissues, and other terms including ‘microtubule’ (GO:0005874), ‘supramolecular polymer’ (GO:0099081), ‘supramolecular fiber’ (GO:0099512), ‘polymeric cytoskeletal fiber’ (GO:0099513), and ‘microtubule cytoskeleton’ (GO:0015630) were enriched in 9 out of 11 tissues. This indicated that genes encoded by the B chromosome might be involved in structural and organizational processes, related to mechanisms associated with the cytoskeleton and microtubules.

### Genes encoded by the B chromosome are probably involved in regulation of meiotic processes

**Fig. 4.**
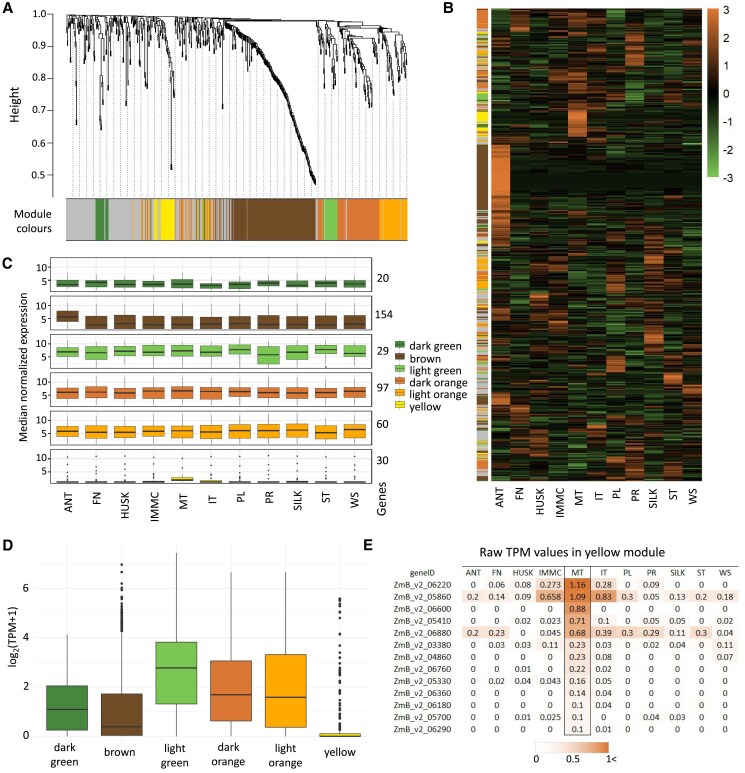
Co-expression analysis of genes encoded on the maize B chromosome, as determined in a B73 inbred line containing two B chromosomes. (A) The clustering of 569 actively expressed genes (total TPM>1 across all samples) located on the B chromosome into co-expression modules according to a weighted gene co-expression network analysis (WGCNA). A total of 390 genes were distributed into six co-expression modules: dark green, brown, light green, dark orange, light orange, and yellow. Grey represents 179 genes with no significant co-expression being identified. (B) Heatmap of expression of genes encoded by the B chromosome across the different tissues according to *z*-scores (key on right). The tissues are listed in Fig. 3 and illustrated in Supplementary Fig. S1. The bar on the left represents the assignment of the assessment of the genes into the modules shown in (A). (C) Box-plot of normalized expression (raw read counts transformed by variance stabilizing transformation) in each tissue of genes encoded by the B chromosome divided into the WGCNA modules. The number of B-genes in each module is indicated. (D) Box-plot of log_2_(TPM+1) values of genes encoded by the B chromosome genes across all the different tissues for each of the WGCNA modules, showing the median values. (E) Raw TPM values of genes assigned to the yellow module across the different tissues, sorted based on the values in meiotic tassels (MT). Only the genes with the highest TPM in MT and TPM>0.1 are shown.

We performed a weighted correlation network analysis using the WGCNA package in R to identify groups of genes that showed similar expression patterns (termed ‘co-expression modules’). The analysis was focused on the 569 genes located on the B chromosome that were considered actively expressed based on a sum of TPM values in all tissue samples >1, and it revealed six distinct modules (Fig. 4A; Supplementary Dataset S7). While the largest module, brown, contained 154 co-expressed genes, the smallest one, light green, contained only 20. There were 179 genes did not exhibit significant correlations with any of the identified modules, and these were placed in the grey module.

Among the six modules, the genes in the brown module showed a notably higher median of normalized expression values in anthers containing mature pollen compared to other tissues (Fig. 4C). This trend was further highlighted in the *z*-score heatmap of B-chromosome gene expression (Fig. 4B), where genes from the brown module clustered together and showed consistently elevated expression specifically in this tissue. A Kruskal–Wallis test indicated very significant differences in expression across tissues (*P*≈1×10^–13^), with subsequent Dunn's *post hoc* test identifying ANT as being significantly different from all the 10 other tissues (*P*≈1×10^–9^–1×10^–10^). The genes forming the light green module had slightly lower (non-significantly) median of normalized expression values in the primary root (PR). The light green cluster also aggregated the genes with the highest expression across all tissues, with a median log_2_(TPM+1) value of 2.77, compared with 0.38–1.68 for most of the other modules, whilst the yellow module had median log_2_(TPM+1)=0 (Fig. 4D). However, genes in the yellow module showed a highly specific expression pattern in tassels containing meiotic pollen (MT), which was significant according to a Kruskal–Wallis test (*P*≈1×10^–4^) and Dunn’s tests for differences in the medians also indicated that this tissue was significantly different to all the others (*P*≈1×10^–3^–1×10^–4^) except for immature tassels (*P*=0.056). Such tissue-specific expression was not observed in any other module.

Whilst the genes in the yellow module had low expression (Fig. 4C), which might be a consequence of the fact that the transcriptome of the reproductive cells represented only a part of the tassel transcriptome), the genes in this yellow module are of particular interest in terms of their possible involvement in B-chromosome-specific inheritance and/or in processes affected by the presence of the B chromosome. Serine/threonine-protein kinases of the Aurora family (represented by ZmB_v2_06220 in Fig. 4E) have previously been described as playing key roles in chromosome segregation in Arabidopsis (Demidov *et al*., 2005) and tobacco (Kurihara *et al*., 2006). Effects of Aurora kinases during oogenic meiosis have also been described in mammals (Øvrebø *et al*., 2015) and in the tunicate *Oikopleura dioica* (Feng and Thompson, 2023). Another interesting gene is *Cyclin-dependent kinase 12* (ZmB_v2_06880), which is involved in meiosis and cell division, not only in plants (Umeda *et al*., 2000; Tank and Thaker, 2011) but also in mice (Ortega *et al*., 2003). However, the most notable gene in the module is *Shortage in chiasmata 1* (ZmB_v2_05860), which is involved in meiosis and has been shown to act in class I crossover formation processes in Arabidopsis (Macaisne *et al*., 2008), humans, and in mice (Guiraldelli *et al*., 2018). Class I crossovers are significantly reduced in Arabidopsis *shoc1* mutants (Macaisne *et al*., 2008). It has previously been shown that the presence of the B chromosome in maize increases the rate of recombination of the A chromosomes in a dosage-dependent manner (Hanson, 1969), and an extra copy of *SHOC1* localized on the B chromosome represents a good candidate for regulating this process.

### The B chromosome influences the expression of genes encoded by A chromosomesin all tissues

**Fig. 5.**
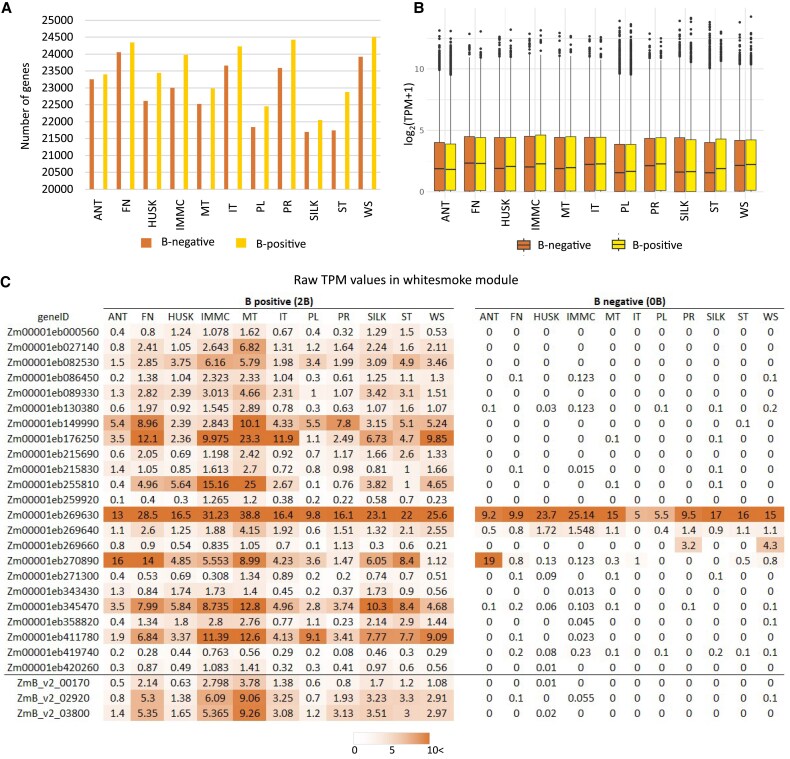
Expression trends of genes encoded by the maize A chromosomes between tissues of the B73 inbred line without B chromosomes (B-negative) and a B73 inbred line containing two B chromosomes (B-positive). (A) Number of A-chromosome genes with TPM>1. The tissues are listed in Fig. 3 and illustrated in Supplementary Fig. S1. (B) Box-plot of log_2_(TPM+1) value of the A-chromosome genes. (C) TPM values of genes assigned with to the ‘whitesmoke’ module in the B-positive and B-negative tissue samples by weighted gene co-expression network analysis (Supplementary Dataset S8). The shading indicates differences in expression, as shown in the key. The last three rows highlight three transcription factors encoded by the B chromosome that were all annotated as transcription initiation factor TFIID subunit 5.

Given that the B chromosome has an impact on the host plant, we examined the effects of its presence on the expression of genes encoded by A chromosomes. We observed a remarkable difference in transcriptomes between tissues originating from plants of the control B73 inbred line without a B chromosome (B-negative) and a B73 inbred line with two B chromosomes (B-positive). The number of genes located on A chromosomes with TPM>1 was higher across all tissues when the B chromosome was present (Fig. 5A). The most notable increase was observed in the leaf tip of the youngest actively growing leaf (ST), with an additional 1132 genes showing expression in B-positive plants compared with B-negative plants, followed by the immature cob (976 genes), primary root (840 genes), and husk (828 genes). As well as an increased number of expressed A-chromosome genes (TPM>1), there was also an increase in their median TPM values in 9 out of 11 tissues analysed (Fig. 5B), suggesting that the presence of the B chromosome was associated with an overall up-regulation of expression of genes on the A chromosomes. However, a slight decrease in median TPM values was observed in anthers with mature pollen and the first internode (FN). In contrast, the increase was most notable in the tissues specific to the ear (IMMC, SILK, HUSK), indicating a significant influence of the B chromosome on the development and function of maize female reproductive organs. It has previously been shown that the B chromosome influences the expression of genes encoded by the A chromosomes in leaves of maize (Huang *et al*., 2016; Shi *et al*., 2022); our findings are consistent with these studies and extend our knowledge to other tissues.

We performed a WGCNA to explore co-expression modules encompassing both genes located on the B and A chromosomes, building on the analysis described above that focused solely on the B chromosome. The WGCNA included genes with total TPM>1 across all samples, ensuring robust expression data, and it identified 169 distinct co-expression modules, with genes clustered based on their expression profiles (Supplementary Dataset S8). Among them, 110 modules contained at least one gene encoded by the B chromosome, four of which contained 15 or more genes.

Ten modules included genes on the B chromosome that were annotated as transcription factors (TFs). The most interesting module, ‘whitesmoke’, contained three transcription factors encoded by the B chromosome (ZmB_v2_00170, ZmB_v2_02920, and ZmB_v2_03800, all annotated as transcription initiation factor TFIID subunit 5) together with 23 genes located on the A chromosomes (Fig. 5C). Notably, the expression of genes located on the A chromosomes was minimal in samples lacking the B chromosome, except for *Zm00001eb269630* and *Zm00001eb270890*. In contrast, the presence of the B chromosome significantly increased the expression of all genes within this module. Based on the functional annotation of the genes with increased expression, they were related to membrane transport (ABC transporters), protein processing (Calreticulin family), metabolism (UTP-glucose-1-phosphate uridylyl transferase), structural and regulatory functions (Armadillo/beta-catenin-like repeat), and stress responses (drought-induced proteins). The increase in expression was highest in tissues associated with reproductive development, specifically in the immature cob (IMMC) and meiotic and immature tassels (MT, IT), which was in correspondence with the expression of TFIID subunits encoded by the B chromosome in the same cluster. These findings highlight the potential regulatory role of the B chromosome in modulating the expression of genes located on both the A and B chromosomes. Most genes encoded by plant B chromosomes were probably introduced by duplication from the A chromosomal complement, as suggested by Blavet *et al*. (2021) and Chen *et al*. (2024). In the case of TFs, such duplications can lead to an imbalance in gene expression. Transcriptional regulation influences chromatin regulation, gene expression, and phenotypic outcomes, and is thus fundamental to organ development and function. Although it is not easy to estimate the effect of TF duplication, a recent study in human cells showed non-linear dose–phenotype relationships (Naqvi *et al*., 2023), suggesting that the effects of B-chromosome-encoded TFs deserve further attention. While it was not our study focus here, previous studies have shown how various numbers of B chromosomes can influence gene expression. For instance, Huang *et al*. (2016) observed that increasing B chromosome number alters the transcription of A chromosomal genes, with more pronounced effects as the B dosage increases. Shi *et al*. (2022) further demonstrated that while the presence of the B chromosome is the main factor modulating the expression of genes on A chromosomes, the expression of B-chromosome genes scales with the B chromosome copy number, reflecting a gene dosage effect. They also found that B chromosome number influences the expression of miRNA and transposable elements located on the A chromosomes. Moreover, the presence of the B chromosome might be stressful for the host plant, resulting in reduced fitness and fertility in some cases (Östergren, 1945; Jones, 1995; Camacho *et al*., 2004; Karafiátová *et al*., 2021). This might be a consequence of the effect of the B chromosome on expression of genes located on the A chromosomes, in addition to a general energy burden during cell division and replication of the supernumerary chromosome.

### Some ribosomal subunits are specifically expressed in the presence of the B chromosome

**Fig. 6.**
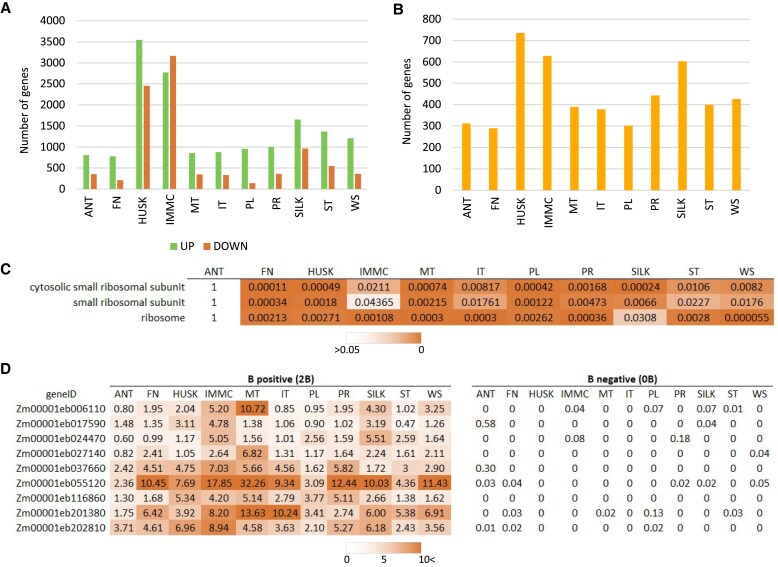
Effect of the maize B chromosome on expression of genes encoded by the A chromosomes. (A) Number of differentially expressed A-chromosome genes in individual tissues. The tissues are listed in Fig. 3 and illustrated in Supplementary Fig. S1. (B) Number of up-regulated A-chromosome genes in tissues of plants of a B73 inbred line containing two B chromosomes (B-positive) that had TPM<1 in the corresponding tissues of plants of the B73 inbred line without B chromosomes (B-negative). (C) Significance values of Fisher’s exact test for gene-set enrichment of the genes shown in (B). Only GO terms significantly enriched in more than half of the tissues are displayed. (D) TPM values of A-chromosome genes with GO term annotations corresponding to the small ribosomal subunit in the tissues of B-positive and B-negative plants.

To better understand the effects of the B chromosome, we performed differential expression and gene-set enrichment analyses to compare the expression of genes located on A chromosomes between B-positive and B-negative plants. This identified 6555 significantly up-regulated and 5580 significantly down-regulated genes [|log_2_(fold-change)|>2, adjusted *P*<0.05] located on the A chromosomes in at least one tissue. There were 257 genes that were significantly up-regulated and 18 significantly down-regulated in all of the tissues. The differential expression was most prevalent in female reproductive organs (Fig. 6A), with the highest number of both up- and down-regulated differentially expressed genes encoded by A chromosomes being observed in the immature cob (up, 2775; down, 3168) and the husk (up, 3546; down, 2459), followed by the silk (up, 1650; down, 965). Other notable effects of the B chromosome on the expression of genes located on the A chromosomes were in the leaf tip of the youngest actively growing leaf (up, 1368; down, 546) and in the whole germinated seedling (up, 1207; down, 362) (Supplementary Dataset S9). These observations were consistent with expression levels of genes located on the B chromosome described above. We next examined up-regulated genes with TPM<1 in the corresponding samples lacking the B chromosome, namely genes that were expressed only in the presence of the B chromosome (Fig. 6B). The highest number of these genes was found in the husk (735), followed by the immature cob (628), and the silk (603) indicating a significant influence of the B chromosome on development and function of maize female reproductive organs.

GSEA of the up-regulated genes located on the A chromosomes with TPM<1 in the corresponding B-negative tissues revealed significant enrichment of 192 GO terms, with the highest number in the husk (71) and the lowest in whole seedlings (39). In all tissues except the anthers, the GO terms ‘cytosolic small ribosomal subunit’ (GO:0022627), ‘small ribosomal subunit’ (GO:0015935), and ‘ribosome’ (GO:0005840) were enriched within the cellular component category (Fig. 6C). For nine genes associated with these GO terms there was either no or very low expression detected in all the B-negative samples, whereas the expression levels of these genes in the B-positive samples were significantly higher, especially for *Zm00001eb055120* (median TPM=10.21 for all tissues), *Zm00001eb201380*, *Zm00001eb202810*, and *Zm00001eb037660* (Fig. 6D). Interestingly, a previous study in Arabidopsis showed that ribosomal remodeling in response to stress involves small ribosomal subunit proteins, which are implicated in the regulation of stress-responsive genes (Fakih *et al*., 2023). Similar roles for ribosomes in abiotic stress responses have been documented in rice (Moin *et al*., 2016) and across plant species more broadly (Dias-Fields and Adamala, 2022). Cheng *et al*. (2025) have recently described this phenomenon in seedlings of B73 inbred maize with different numbers of B chromosomes by studying the physiological responses of the seedlings to the B chromosome, with the results indicating possible increases in stress tolerance. In contrast, it has also been considered that B chromosomes have mostly negative effects on the host plant (Östergren, 1945). The up-regulation of genes related to ribosomes on the A chromosomes that we observed in plants possessing the B chromosome could go some way to proving an explanation, as it suggests a potential adaptive response, whereby ribosome-related pathways are activated to mitigate the stress associated with the presence of the B chromosome.

## Conclusion

Through systematic transcriptional analyses across 11 diverse tissues of maize plants with and without the B chromosome, our study has revealed expression of genes encoded by the B chromosome at all developmental stages in the life cycle. Co-expression analysis of genes encoded by the B chromosome indicated an effect on meiosis and identified a possible regulator involved in increased recombination frequency mediated by the B chromosome. A striking effect of the presence of the B chromosome on the expression of the A chromosomal complement was found. Transcription factors from the B chromosome seem to control the expression of a group of genes of the A chromosome set, and the presence of the B chromosome stimulates the expression of several proteins of the small ribosomal subunit that might be involved in the response to stress conditions. These findings advance our knowledge of supernumerary chromosomes and pave the way for exploring their broader roles in plants.

## Supplementary Material

eraf337_Supplementary_Data

## Data Availability

Raw sequence reads are available at the European Nucleotide Archive (https://www.ebi.ac.uk/ena/browser/home) under accession numbers PRJEB86401 (whole-genome sequencing short reads, Oxford Nanopore, Hi-C sequencing, plus the reference sequence and gene annotations) and PRJEB86173 (RNA-seq).

## Supplementary data

The following supplementary data are available at *JXB* online.

Fig. S1. Illustration of the tissues examined in the transcriptome analysis.

Fig. S2. Use of optical maps for gap-closing in the B chromosome pseudomolecule.

Fig. S3. Sequence alignment between the B chromosome pseudomolecule generated in this study and that of Blavet *et al*. (2021).

Fig. S4. Gene-set enrichment analysis of genes encoded by the B chromosome in the different tissues.

Table S1. Summary statistics of the ONT sequencing.

Table S2. BUSCO assessment of the completeness of the assembly.

Table S3. Assembly statistics after Canu assembly and after Hi-C scaffolding.

Table S4. Optical map statistics.

Dataset S1. Information about the annotated genes.

Dataset S2. Comparison between the annotated genes generated by Blavet *et al*. (2021) and by the current study.

Dataset S3. Results of homology searches between the protein sequences generated in this study and *Z. mays* RefGen_v5 protein sequences and proteomes from the *Magnoliopsida* class.

Dataset S4. Gene ontology enrichment analysis.

Dataset S5. TPM values for genes encoded by the A and B chromosomes.

Dataset S6. Gene-set enrichment analysis of genes with TPM>1 encoded by the B chromosome.

Dataset S7. Assignment of genes encoded by the B chromosome to WGCNA modules.

Dataset S8. Assignment of genes encoded by the A and B chromosomes to WGCNA modules.

Dataset S9. Results of differential expression analysis in the different tissues between plants with and without the B chromosome.
