# Supplementary material for: The maize B chromosome shapes the transcriptome throughout the entire plant life cycle
Source: J Exp Bot. 2025 Jul 29;76(22):6810–25. doi: 10.1093/jxb/eraf337 (PMC12675260; doi:10.1093/jxb/eraf337)
Supplement: eraf337_Supplementary_Data [file eraf337_supplementary_data.zip › eraf337 NEW - Supplementary Data.pdf]

Maize B chromosome shapes the transcriptome throughout the entire plant life cycle

Lucie Hloušková[1,2], Zuzana Tulpová[1], Radim Svačina[1], Kateřina Holušová[1], Petr Cápal[1], Pavla Navrátilová[1], Miroslava Karafiátová[1] and Jan Bartoš[1*]

[1]Institute of Experimental Botany of the Czech Academy of Sciences, Centre of Plant Structural and Functional Genomics, Šlechtitelů 31, Olomouc 779 00, Czech Republic

[2]Department of Cell Biology and Genetics, Palacký University, Šlechtitelů 27, Olomouc 779 00, Czech Republic

* Corresponding author: Jan Bartoš; bartos@ueb.cas.cz

Supplementary Data

Dataset S1. Information about the annotated genes.

Dataset S2. Comparison between the annotated genes generated by Blavet *et al.* (2021) and by the current study.

Dataset S3. Results of homology searches between the protein sequences generated in this study and *Z. mays* RefGen_v5 protein sequences and proteomes from the *Magnoliopsida* class

Dataset S4. Gene Ontology enrichment analysis.

Dataset S5. TPM values for genes encoded by the A and B chromosomes.

Dataset S6. Gene-set enrichment analysis of genes with TPM>1 encoded by the B chromosome.

Dataset S7. Assignment of genes encoded by the B chromosome to WGCNA modules.

Dataset S8. Assignment of genes encoded by the A and B chromosomes to WGCNA modules.

Dataset S9. Results of differential expression analysis in the different tissues between plants with and without the B chromosome.

Supplementary Tables

Table S1: Summary statistics of the ONT sequencing. A - Reads generated using Ultra-long Sequencing Kit. B - Reads generated using Rapid Sequencing Kit. C - Reads generated in total.

**A - Ultra-long library**		**B - Rapid library**	
Number of reads	4 920 109	Number of reads	3 454 646
Read length N50	56 822	Read length N50	11 393
Total bases	83 040 996 385	Total bases	19 903 051 780
Mean read length	16 877.9	Mean read length	5 761.2
Mean read quality	11.4	Mean read quality	11.2
**C - In total**			
Number of reads	8 374 755		
Read length N50	42 688		
Total bases	102 944 048 165		
Mean read length	12 292.2		
Mean read quality	11.3		

Table S2: BUSCO assessment of the completeness of the assembly. C – complete genes, S – complete and single-copy BUSCOs, D – complete and duplicated BUSCOs, F – fragmented BUSCOs, M – missing BUSCOs. 01 – the initial Canu assembly of ONT reads. 02 – the initial assembly after two iterations of NextPolish polishing using Illumina reads. 03 – the assembly after Hi-C scaffolding. 04 – the assembly after closing the gaps. 05 – final assembly after two iterations of NextPolish polishing using ONT reads and two iterations of NextPolish using Illumina reads.

01 – canu assembly	C:96.2%[S:68.2%,D:28.0%],F:1.1%,M:2.7%
02 – after NextPolish	C:98.1%[S:66.7%,D:31.4%],F:0.3%,M:1.6%
03 – HiC scaffolding	C:98.1%[S:67.0%,D:31.1%],F:0.3%,M:1.6%
04 – gap closing	C:98.1%[S:67.0%,D:31.1%],F:0.3%,M:1.6%
05 – final assembly after NextPolish	C:98.3%[S:66.1%,D:32.2%],F:0.2%,M:1.5%

Table S3: Assembly statistics after Canu assembly and after Hi-C scaffolding.

**Canu assembly**	
Number of contigs	4290
Contig N50	3 651 901
Number of B-originated contigs	56
B-originated contig N50	7 747 378
**After Hi-C scaffolding**	
Number of B-originated scaffolds	61
Longest B-originated scaffold	scaffold_10: 116 793 091
B-originated scaffold N50 (without scaffold_10)	569 359

Table S4: Optical map statistics.

**Filtered molecules (MaxInt2000, >150 kbp)**	Total length	947.5 Gbp
	Molecule N50	255.5 kbp
	Molecule coverage	421 x
**Optical map assembly (complete)**	No. of contigs	1 170
	Total length	3.3 Gbp
	Contig N50	49.5 Mbp
	Average contig length	2.8 Mbp
**Optical map assembly (B-originated)**	No. of contigs	594
	Total length	288.6 Mbp
	Contig N50	880.5 kbp
	Average contig length	485.9 kbp

Supplementary Figures

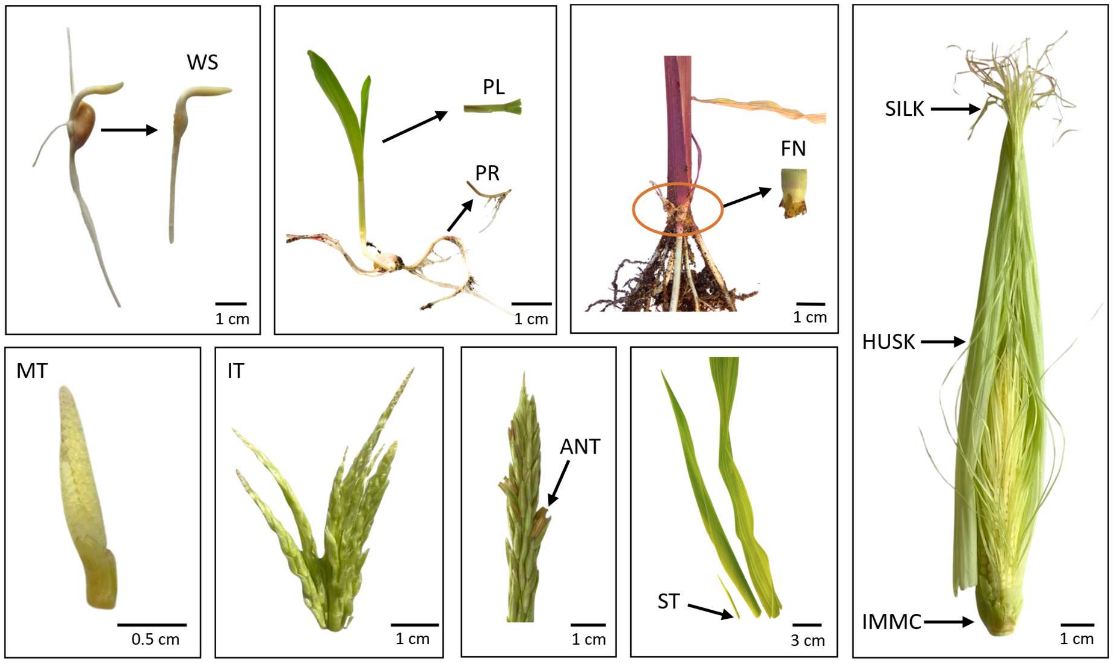

Fig. S1: Illustration of the tissues examined in the transcriptome analysis: whole germinated seedling (WS), primary root (PR), and first leaves (PL) at the one-leaf stage (V1); first stem node (FN) and leaf tip of the youngest actively growing leaf (ST) at the five-leaf stage (V5); meiotic tassel (MT); immature tassel (IT); anthers with mature pollen (ANT); unpollinated immature cob (IMMC); and the husk (HUSK) and silk (SILK) of the unpollinated immature cob.

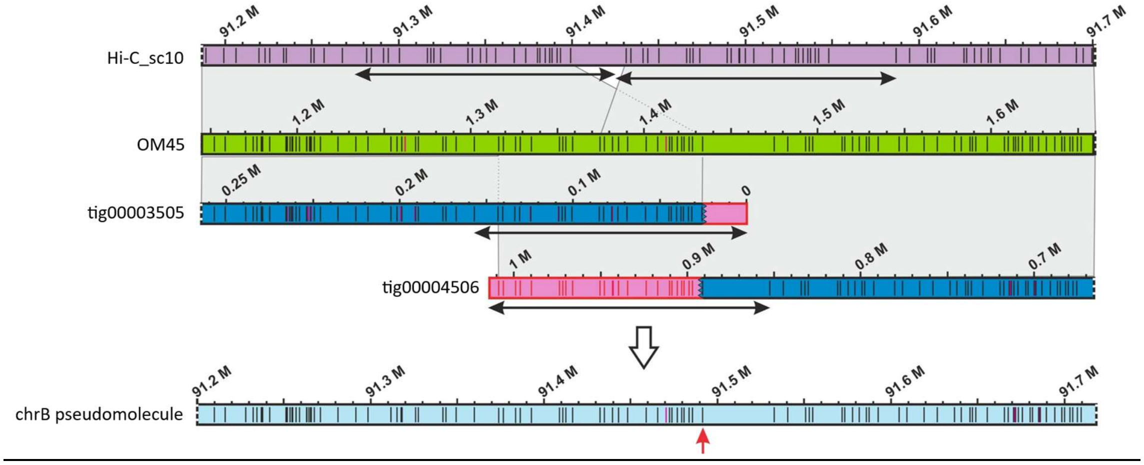

Fig. S2: Use of optical maps for gap-closing in the B chromosome pseudomolecule. Alignment of contigs of assembly generated by Canu software (dark blue bars) to the optical map (green bar) and Hi-C scaffold (purple bar) revealed duplicated region in Hi-C scaffold (highlighted with double-way arrows). The duplicated part is also located at the ends of contigs 3505 and 4506 (highlighted with pink). Overlapping parts were cut (jagged vertical line) to avoid generation

of artificial duplication and contigs were merged to create contiguous region in chromosome B pseudomolecule (light blue bar). Joining site in the chromosome B pseudomolecule is highlighted with a red arrow. Vertical lines represent DLE sites in the optical map, Hi-C scaffold, contigs and pseudomolecule. Note the correspondence of the final pseudomolecule with the optical map.

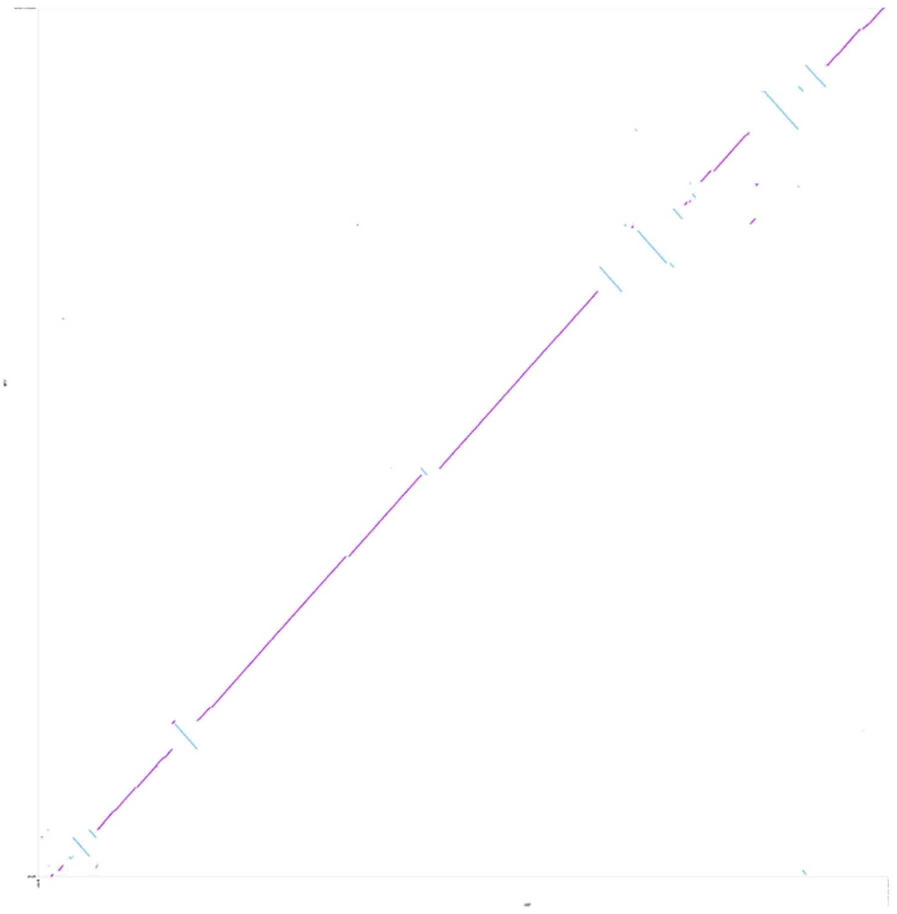

Fig. S3: Sequence alignment between the B chromosome pseudomolecule generated in this study and that of Blavet *et al.* (2021). X axis - Maize_Bchr_v2 (this study). Y axis - Zm-B73_B_CHROMOSOME-MBSC-1.0 (Blavet *et al.*, 2021).

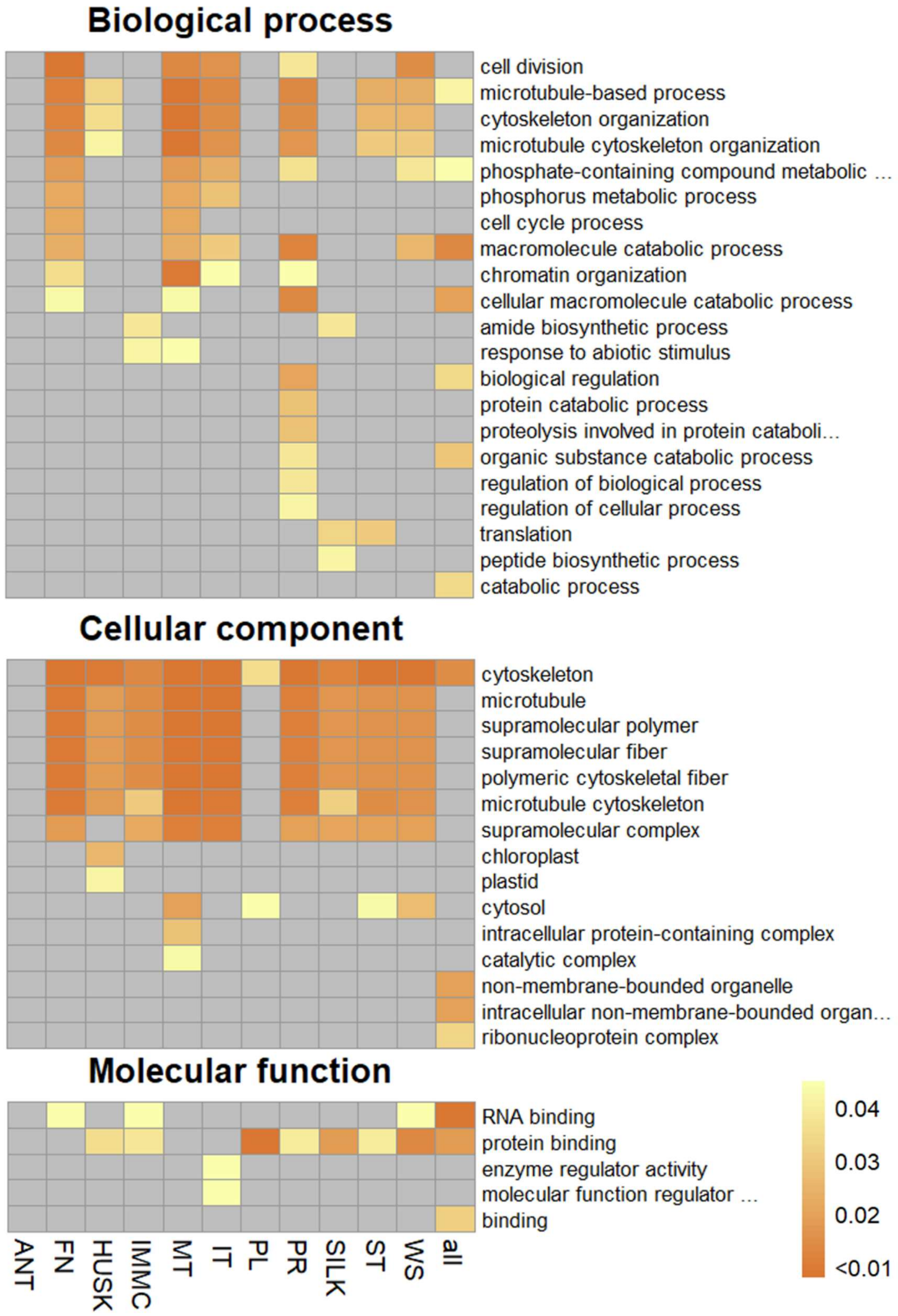

Fig. S4: Gene-set enrichment analysis of genes encoded by the B chromosome in the different tissues. Genes were filtered: TPM > 1, Fisher's exact test p-value < 0.05. "All" category corresponds to genes expressed across all tissues. Colour key corresponds to p-value.
